# Analysis of biomedical data with multilevel glyphs

**DOI:** 10.1186/1471-2105-15-S6-S5

**Published:** 2014-05-16

**Authors:** Heimo Müller, Robert Reihs, Kurt Zatloukal, Andreas Holzinger

**Affiliations:** 1 Institute of Pathology, Medical University of Graz, Auenbruggerplatz 2, A-8036, Graz, Austria; 2 Institute for Medical Informatics, Statistics and Documentation, Medical University of Graz, Auenbruggerplatz 2, A-8036, Graz, Austria; 3 Institute of Information Systems and Computer Media, Graz University of Technology, Inffeldgasse 16c, A-8010, Graz, Austria

**Keywords:** Visualization, Interactive Knowledge Discovery, Glyphs, Semantic Zoom

## Abstract

**Background:**

This paper presents multilevel data glyphs optimized for the interactive knowledge discovery and visualization of large biomedical data sets. Data glyphs are three- dimensional objects defined by multiple levels of geometric descriptions (levels of detail) combined with a mapping of data attributes to graphical elements and methods, which specify their spatial position.

**Methods:**

In the data mapping phase, which is done by a biomedical expert, meta information about the data attributes (scale, number of distinct values) are compared with the visual capabilities of the graphical elements in order to give a feedback to the user about the correctness of the variable mapping. The spatial arrangement of glyphs is done in a dimetric view, which leads to high data density, a simplified 3D navigation and avoids perspective distortion.

**Results:**

We show the usage of data glyphs in the *disease analyser *a visual analytics application for personalized medicine and provide an outlook to a biomedical web visualization scenario.

**Conclusions:**

Data glyphs can be successfully applied in the *disease analyser *for the analysis of big medical data sets. Especially the automatic validation of the data mapping, selection of subgroups within histograms and the visual comparison of the value distributions were seen by experts as an important functionality.

## Background

Professionals in the biomedical domain are confronted with increasing masses of data, which require efficient and user-friendly solutions and the development of methods to assist them in knowledge discovery to identify, extract, visualize and understand useful information from these large amounts of data [1]. The trend towards personalized medicine has resulted in a mass of clinical, laboratory and genome-scale

data and moreover, most data models are characterized by complexity, which makes manual analysis very time-consuming and frequently practically impossible [2]. The major challenge is: How can an expert find knowledge in these terabytes of complex data? For example, to successfully search for novel hypotheses in large datasets, we must look for unexpected patterns and interpret evidence in ways that frame new questions and suggest further explorations[3]. Consequently, methods from Knowledge Discovery and Visual Analytics methods may help us to

• Overview large data sets as the human visual sense is optimized for parallel processing

• Connect the global view with detail information

• Provide different contextual views (e.g. expert versus common user)

• Deal with inhomogeneous data sets and broad range of data quality.

As one solution to these goals, we developed a set of validated glyphs for interactive exploration of biomedical data sets. With the ability to work with different level of details, to arrange and order the glyphs in space and to synchronise different visualizations through coordinated multiple views (CMV) [4], an expert can in the truest sense of the word, travel through his data space.

Jacques Bertin's book Sémiologie graphique, published in 1967 (English translation 1987 by J. Berg), provides the foundation for the analysis of visual elements to display qualitative or quantitative data [5]. Bertin's practical experience as a cartographer led him to the question how to find rules to build proper graphics. His study of signs together with their "grammatical" rules is based on a clear and logical symbol scheme in which symbols can be varied referring to visual variables. Visual variables include size of elements, their shape, orientation, brightness color, texture and position. Bertin called these attributes also retinal variables, because they describe the quality characteristics of the human perception, in contrast to a technical description of a graphical element. Actually, this leads to semiotics - and we view informatics as semiotics engineering [6], because it is interesting to observe that the three main goals of informatics (correctness of algorithms, efficiency of programs, and usability of software systems) turn out to be nicely related to the three semiotic dimensions [7]: 1) Correctness is a matter of syntax to be answered by considering formal aspects only [8]; 2) Efficiency is a matter of semantics related to the object world [9]; and 3) Usability, taking interest and motivation of the end user into account [10]; being our basic assumptions for the following details:

A **visual variable **is characterized according to Bertin by the kind of scale (nominal ordinal) and the length of the visual variable. The length of a variable is the number of distinguishable values that can be perceived by a viewer (for example how many shades of grey or different hue values can be differentiated) Choosing different visual variables for representing the same data variable greatly influence the perception and understanding of the glyph. It is therefore important to know and appropriately map data variables to visual variables in the design of a glyph.

Our approach will make use of visual variables to describe the perceptual properties of a glyph. Ropinski & Preim (2008) and Ropinski, Oeltze & Preim (2011) [11], [12] describe glyph-based visualization techniques in medical visualizations and give a glyph taxonomy together with guidelines for the usage of glyphs. Ward (2002) [13] describes a taxonomy of glyph placement strategies, were he distinguishes between data-driven and structure-driven approaches. He also describes strategies to avoid overlapping problems and proposes a spacefilling layout for structured data.

A very specific type of glyphs was introduced by Chernoff (1973): the so-called Chernoff faces [14]. Chernoff faces are 2D glyphs, which employ human's ability to recognize faces and small changes in facial characteristics. However the effectiveness of this form of visualization is still being debated in the scientific community [15], [16].

Kraus & Ertl [17] present in a more technical approach a system for glyph generation (with minimal user interaction) which has been used in a visualization tool in the automotive industry.

An overview about the state of the art in the visualization of multi-variate data is given by Peng & Laramee (2009) [18] as well as Bürger & Hauser (2007), where they discuss how different techniques take effect at specific stages of the visualization pipeline and how they apply to multi- variate data sets being composed of scalars, vectors, and tensors. Moreover they provide a categorization of these techniques in the aim for a better overview of related approaches

[19], with an update published 2009 [20]. Visual data exploration methods on large data sets were described by several authors, and particularly Keim (2001) [21], Hege et al. (2001) [22], Fayyad, Wierse & Grinstein (2002), [23], Fekete & Plaisant (2002) [24], and Santos & Brodlie (2004) [25] provide a good introduction to this topic. A recent state-of-the-art report on glyph based visualization and a good overview on theoretic frameworks, e.g. on the semiotic system of Bertin, was given by Borgo et. al. (2013) [26].

An interesting application of glyphs for a visual analytics approach for understanding biclustering results from microarray data has been presented by Santamaria, Theron & Quintales (2008), [27] and another one by Gehlenborg & Brazma (2009), [28] and Helt et al (2009), [29] and a recent work by Konwar et al (2013), [30].

The closest work to use glyphs with an adaptive layout is the work of Legg et al. (2012) [31] in the application domain of sport analysis. Here the data space is event based, and the adaptive layout strategy is focused on overlapping events with so called "macro glyphs", which combine several glyphs into one. In the "macro glyph" approach only scaling and no level of detail (LoD) suitable for different screen spaces are applied. In the evaluation phase expert interviews at the work environment level. based on methods described by Tory & Möller (2004) [32] and Plaisant (2004) [33] were done.

## Methods

### Data glyphs

**Figure 1:**
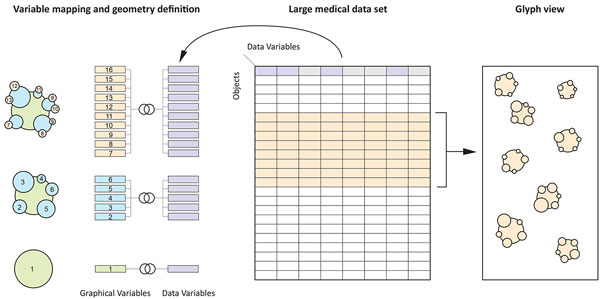
**Multilevel Data Glyphs (figure1.pdf)**. The overall principle of multilevel data glyphs.

Data glyphs are composed by (i) a mapping of data variables to visual primitives, e.g. lines, shapes, fonts. Each of the visual primitives is described by its visual capabilities according to Bertin's visual variables (ii) combination of the visual primitives into compound shapes, (iii) organization of he compound shaped into level of details (LoD) and (iv) spatial positioning and rendering algorithms, see Figure 1.

Our previous work [34,35] in biomedical visualization resulted in an upper bound of 16 attributes for the highest level of detail. This number is given be the attribute set in a pathological finding, which is composed of patient information (age, sex, year of birth, year of death, cause of death, disease free survival), the pathological finding (organ, size of the tumor, lymph nodes staging, metastasis staging, grading, receptor state ) and surgery attributes (origin of the sample, year of surgery, doctor, type of sample). In order to unveil hidden relations by the recognition of unexpected patterns, as many variables as possible should be integrated within the rendering of one glyph. 2D glyph designs are usually limited to up to 5 data variables, therefore we chose the approach to model data glyphs as 3D objects. This results on the one hand a high information density but on other hand we face the problems of occlusion, perspective distortion and complex navigation and orientation in 3D space. Usability tests with very first prototypes have indicated that glyphs placement in 3D space using a perspective projection and the possibility to freely move within this space was overly burdensome for almost all users, especially for medical experts. To avoid the problems described above, we restricted the 3D space to 2.5D or to a ¾ perspective view by applying dimetric (near isometric) projection grid, well known from technical illustrations and from some very successful simulation games of the 1990s (e.g Civilization ) In a diametric projection grid data glyphs do not change size as they are moved, so no re-rendering of a glyph is necessary to simulate a "¾ perspective view. With a dimetric projection grids also specific performance optimization strategies, e.g. bitmap caching and selection highlighting can be easily applied.

### Level of detail

As we want visualize several millions data elements in the smallest level of detail, the screen size of a glyph can be as small as one pixel. Therefore only the visual variable "value" (from light to dark) or "color" (changes in hue at a given value) can be the starting point. Note: If the maximal number of elements to be visualized is in the range of several 10.000 elements, we can also choose the visual variable shape as starting point. To achieve well-graduated levels of details and visually smooth transition between leves we rely at the principle that the dominant visual variable of level n is also the strongest visual variable in level n+1.

**Figure 2:**
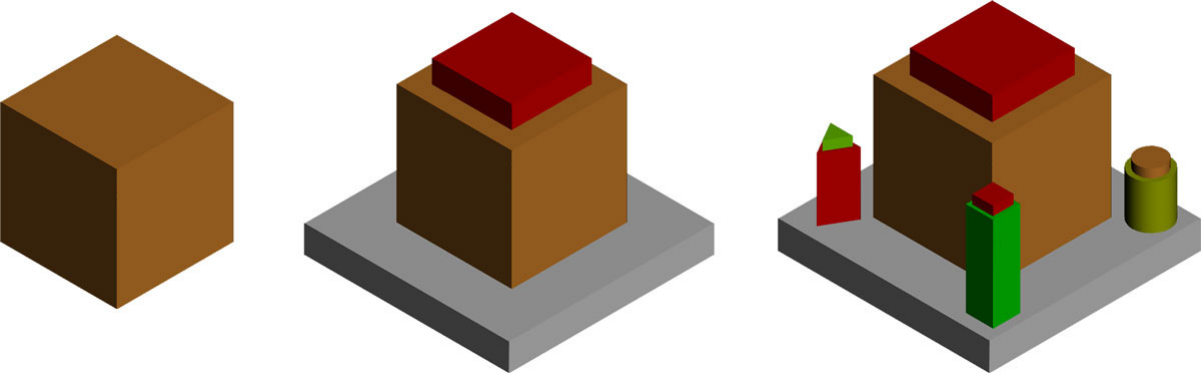
**Cubic Glyph (figure2.png)**. Example of a cubic glyph design

In previous work [35] several glyph designs were developed, but not evaluated. A systematic evaluation with medical expert (n = 12) resulted in a very clear results, (10/12) were in favour of "cubic glyphs", with the two main arguments: all graphical elements are necessary and useful (no disturbing visual variables) and the transition between level is naturally (the form of a rectangular cubic glyph corresponds well to a square pixels). An example cubic glyph can be seen in Figure 2, the corresponding visual variables are summarized in Table 1. The 3 levels of the cubic glyphs are: (i)

**Table 1:** Visual Variables of the Cubic Glyph

	**Visual Variable**	**Level**	**Type**	**Scale**	**Length**

1	primary color	1	color	nom/ord	short
2	height main cube	2	geometry size	ordinal	long
3	color cap	2	color	nom/ord	short
4	color base	2	color	nom/ord	short
5	size cap	2	geometry shape	ordinal	medium
6	height cap	2	geometry size	ordinal	long
7	shape cap	2	geometry size	ordinal	short
8	height west-element	3	geometry size	ordinal	long
9	color west-element base	3	color	nom/ord	short
10	color cap east-element	3	color	nom/ord	short
11	height east -element	3	geometry size	ordinal	long
12	color east -element base	3	color	nom/ord	short
13	color cap east -element	3	color	nom/ord	short
14	height south-element	3	geometry size	ordinal	long
15	color south-element base	3	color	nom/ord	short
16	color cap south-element	3	color	nom/ord	short

The *pixel level*, were one data attribute determines the color of the glyph either by direct mapping, a color gradient or a custom (algorithmic) mapping. This color will be the dominant color also in all higher levels. The pixel level is applied, when the screen size of a glyph is below 2x2 pixels. At the pixel level a user can interact (filter, group, arrange, cluster) with several million glyphs. (ii) In the *iconic level *we add 6 additional visual variables. At the iconic level a user can interact (filter, group, arrange, cluster) with several thousands elements. And finally (iii) the detail level, were we add 9 geometric primitives to the data glyph, which results in an overall number of maximal 16 data attributes mapped to a single glyph. A glyph is rendered in the detail view when its screen size is greater then 64x64 pixels. At the detail level a user can interact (filter, group, arrange, cluster) with several thousands elements

### Glyph Placement

According to the taxonomy given by Ward [13] we support:

• User driven placement, in which case the user determines the position of a glyph through interaction tasks (selection, filtering, movement, grouping)

• Data driven placement, in which case data values are used to specify the location of the glyph. Our placement strategy supports value discretization and jittering strategies for the placement in an dimetric projection grid,

• Structure driven placement, in which case relationship between data points determines the location of a glyph. We support structure directly derivable from the data values, e.g. grouping glyph representing cancer cases by year of surgery, sex and cancer staging, and glyph placements determined by interactive ant clustering algorithm.

**Figure 3:**
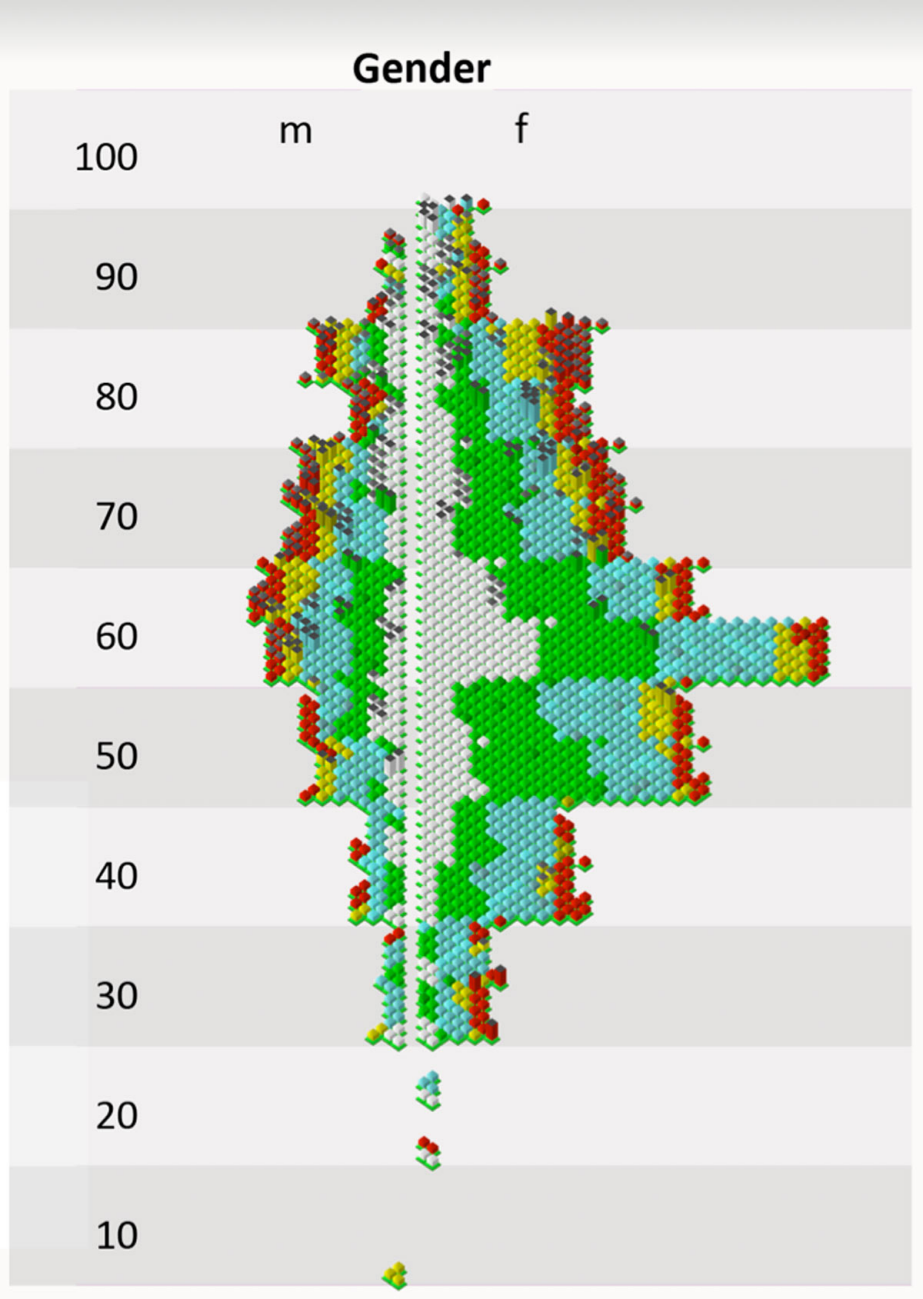
**Cubic glyphs arranged in an age pyramid (figure3.png)**. Spatial arrangement of iconic glyphs in a age pyramid. All male thyroid cancer patients are on the left side and female patients on the right side. The vertical position of a glyph is determined by the patients' age and the horizontal position by the T-staging. The T-staging is also the variable used in the mapping of the primary level.

Figure 3 shows a spatial arrangement of glyphs in iconic level in an age pyramid. All male patients are on the left side and female patients on the right side. The vertical position of a glyph is determined by the patients age and the horizontal position by the size of the tumor given by the T-staging of the pathological finding [36]. The T-staging is also the variable used in the mapping of the primary level.

### Mapping validation

A data glyph can be configured through the mapping of data variables to the parameters of its geometric primitives. This is on the one hand a very powerful tool, as the user can map any data attribute to any geometric parameter, and even change the mapping on the fly, on the other hand its also crucial, because the great flexibility could easily lead to faulty mappings (e.g. mapping a nominal variable to the position of a geometrical primitive) and in succession to misinterpretations of the visualizations results. In order to avoid those mismatches we provide an automatic validation of the variable mapping.

In the automatic validation, we compare meta information about data variables - scale of measurement (discrete, continuous, categorical, ordinal, interval, nominal) and the number of distinct values - to the visual capabilities of the glyph elements. The verification is done according to the following rules:

The *shape *of a geometric primitive is purely nominal and should therefore never be mapped to ordinal data values. However we can recognize a almost infinite variety of shapes (the shape variable is "very long").

The perceptual variable *color (hue) *is a nominal variable, even though the wavelength of light assigns an ordering to colors, the human perceptual system takes no notice of it. There is some cultural ordering imposed on hue (red is "hotter" than blue), but it is weak because not all hues are related. A non-color deficient person can distinguish between seven and ten million different colors. However, color is a deeply subjective attribute, and therefore not more than 10 to 20 carefully chosen color values should be used in color mapping. A great tool for carefully designed colormaps, which e.g. provides "colorblind safe" suggestions, can be found at colorbrewer2.org[37]

Value (the brightness of an element) and the texture (with respect to the grain size of the texture) are ordered and can be mapped to an ordinal scale. Value and texture are short variables, i.e. roughly 10 values can be distinguished in an effective way.

The position of a glyph can be mapped to ordinal values, and is a very fine-grained (long) variable. The size of a geometric primitive, or even of the whole glyph element can also be mapped to ordinal values, but it is "shorter" than the position variable.

Finally the orientation of a geometric primitive can be mapped to an ordinal data value, but this is a very short viusal variable, i.e. only very few different orientations can be perceived.

## Results

We use multilevel data glyphs in the *disease analyser*, a visual analytic application for the interactive exploration of a database containing approximately 1,4 million cancer cases. Each record describes a comprehensive diagnosis of a cancerous (malignant) tumor case. The most used variables are patient age and sex, the ICDN classification, the TNM staging, grading receptor states and information about the time under risk, disease free survival and overall survival together with surgery information.

**Figure 4:**
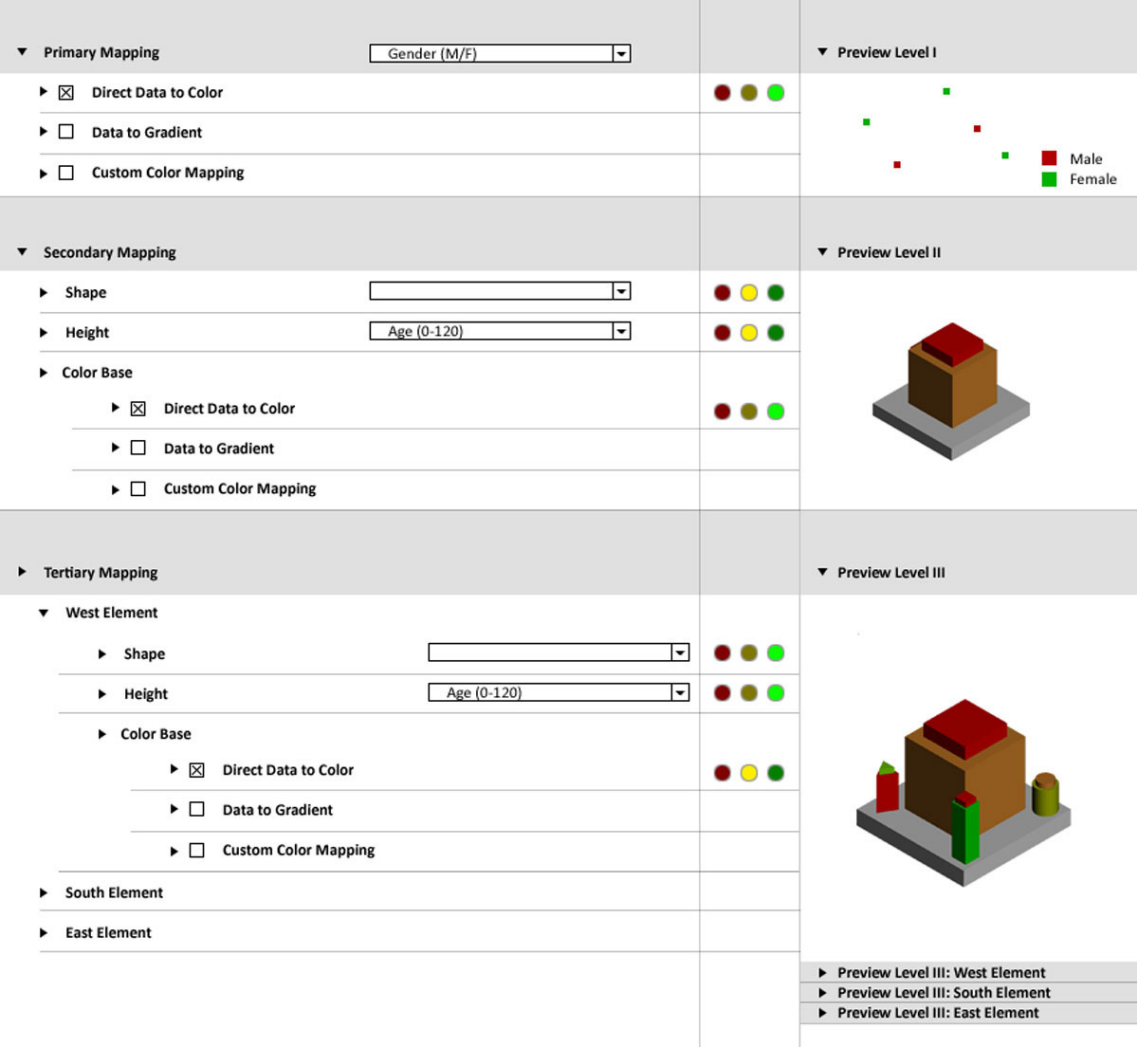
**Variable Mapping (figure4.png)**. Mapping of the data variables to visual variables of the data glyph. A "traffic light" visualization indicates the validity of the mapping.

Figure 4 shows the mapping of the data variables to visual variables of the data glyph. In this interface we use "traffic light" indicator to show the validity of the mapping.

• Green: All data scales fits to the scale of corresponding visuals variable the length of all visual variables is equal/greater then the corresponding distinct data values.

• Yellow: All data scales fits to the scale of visuals variables and the length of some visual variable is smaller then the number of corresponding distinct data values.

• Red: There is a mismatch (minimal one) attribute scale and the scale of the corresponding visual variable.

**Figure 5:**
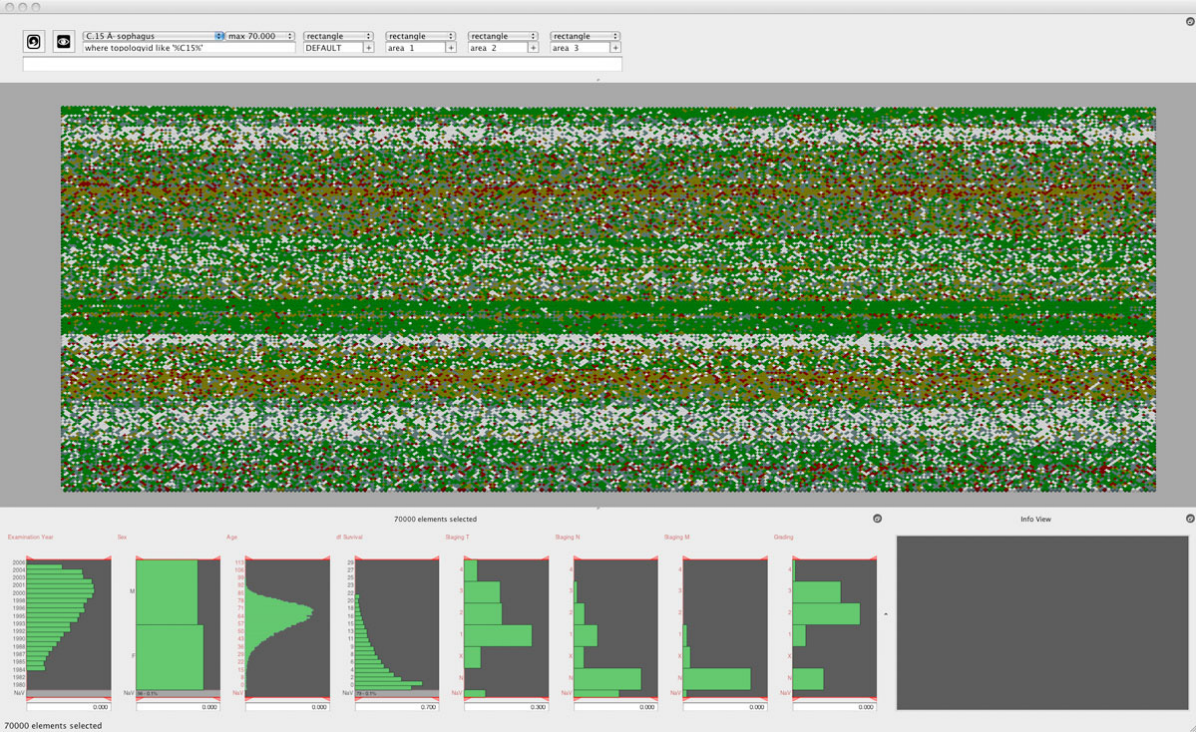
**70000 *cancer cases randomly selected from the disease database *Distribution of Teaching Types (figure5.png)**. Approx. 70.000 randomly selected entities from the disease database. For this number of elements we use the pixel level for the data glyph, i.e. only the color of the glyph is given by its primary mapping, the T-staging. The spatial position of the glyphs in the starting view is just determined by the ordering of the cases within the database. In the lower part of the *disease analyser *histograms of the variables used in the glyph mapping are shown

Figure 5 shows approx. 70.000 randomly selected entities from the disease database. We took this high number of cases to get a proportionate sampling for all organs. For this high number of cases glyphs are rendered in the pixel level, i.e. the T-staging (size of the tumor) maps to the color of the. The spatial position of the glyphs in the starting view is just determined by the ordering of the cases within the database.

**Figure 6:**
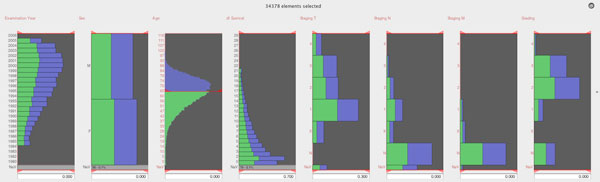
**Selection of Subgroups (figure6.png)**. Histograms for the examination year, sex, age, disease free survival, T-staging, N-staging, M-staging and the grading. The histogram view shows the value distribution of the selected cases (green area) in relation to the overall distribution of cases (blue area). See also the additional file suppl_linked_sliders.mov

In the lower part of the *disease analyser *histograms of the attributes of cancer findings are shown. Figure 6 shows the histograms for the examination year, sex, age, disease free survival, T-staging, N-staging, M-staging and the grading. In the next step an expert can divide cases into two subgroups, in our example by patient age. The histogram view shows the value distribution of the selected cases (green area) in relation to the overall distribution of cases (blue area). The specification of subgroups (filtering by value ranges for each attribute) together with glyph highlighting and re-ordering can be done in real-time. The interface for this filtering task is embedded into the histograms (red sliders). See the supplement video "linked histogram sliders.mov".

**Figure 7:**
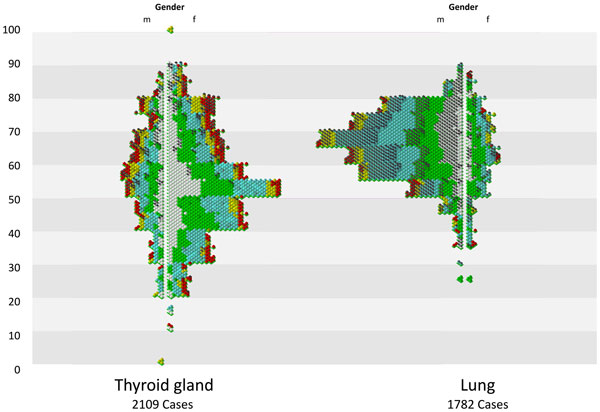
**Comparison of 2109 thyroid and 1782 lung cancer cases**. Selection of Subgroups (figure7.png) 2109 thyroid cancer cases and 1782 lung cases, both arranged in an age pyramid. The relatively low number of cases result in bigger glyphs sizes, therefore the rendering of the glyphs is done at the iconic level.

**Figure 8:**
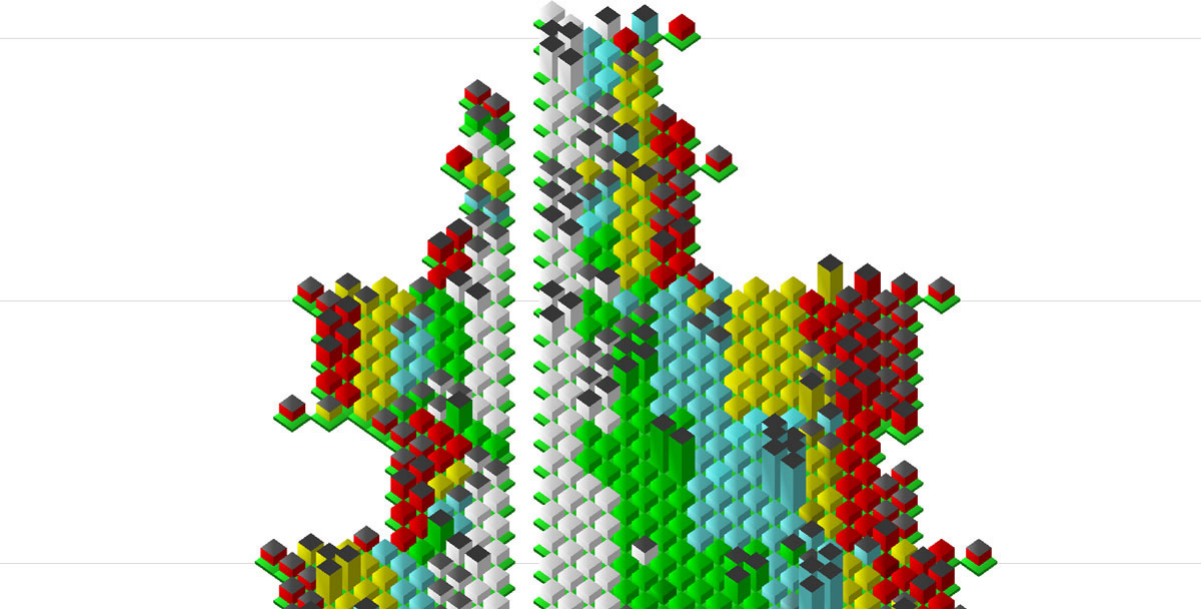
**Detail view of the thyroid cancer visualization with iconic glyphs (figure8.png) Zoom-in of the visualization of figure 10**.

In the next example an expert compares cancer cases for different organs. Figure 7 shows 2109 thyroid cancer cases and 1782 lung cases, both arranged in an age pyramid. The relatively low number of cases result in a screen size, therefore the rendering of the glyphs is done at the iconic level. In Figure 8 we see the iconic glyphs in a zoomed state (upper part of the thyroid cancer). The visualization shows difference in gender distribution (much more men have lung cancer), difference in mortality (much more black caps in lung cancer then in thyroid cancer), high overall survival of a subgroup in thyroid cancer (glyph without black cap). Beside of the overview and comparison of two medium size groups, outliers can be identified easily (thyroid cancer cases with age of 0 and 100 years, which are data input errors).

**Figure 9:**
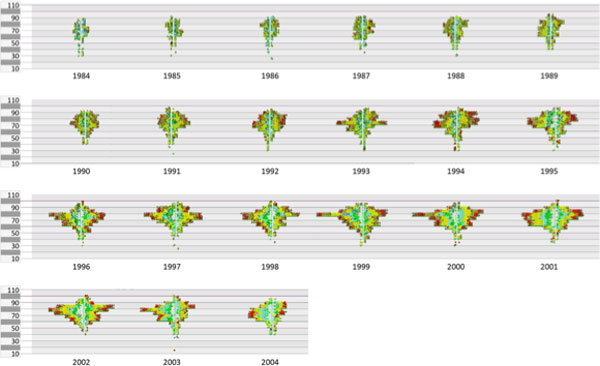
**11.000 colon cancer cases grouped by the examination year**. The visualization is done at the pixel level (figure9.png). 11.000 colon cancer cases, grouped by the examination year (1984 to 2004). For each year the glyphs are arranged in an age pyramid. A medical expert can overview a very large number of cases and recognise in a trend analysis several aspects, e.g. the increase of cases, shift in age distribution, increase in small tumors through by early warning programs, two outliners in the 1999/2000 for male patients in the age group 75-80 (no explanation yet).

Figure 9 shows about 11.000 colon cancer cases rendered in the pixel level. The glyphs are grouped by the examination year (1984 to 2004). For each year the glyphs are arranged in an age pyramid. Here a medical expert can overview a very large number of cases and recognise in a trend analysis several aspects. For colon cancer cases the following observations were made. (i) There is a strong increase of cases, (ii) a shift in age distribution and increase in small tumors through by early warning programs can be clearly seen and (iii) two outliners in the 1999/2000 for male patients in the age group 75-80 were identified, with no explanation yet.

**Figure 10:**
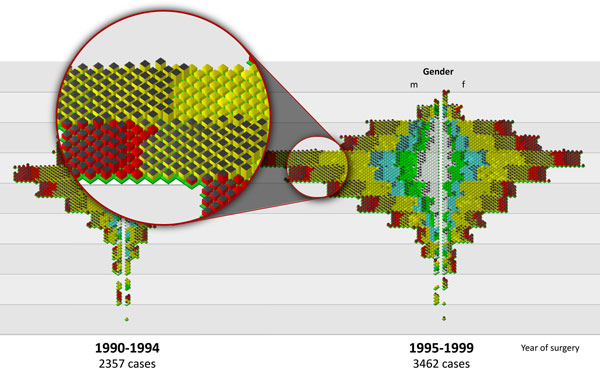
**Colon cancer age pyramid of 5-year periods**. The visualization is done with iconic glyphs (figure10.png). Regrouping of the colon cancer cases to 5 year time periods. In the iconic view we can see additional information about the mortal state and disease free survival period of a patient.

Figure 10 shows the regrouping of the colon cancer cases to 5 year time periods. In the iconic view we can see additional information about the mortal state and disease free survival period of a patient. In the period (1995-1999) it was clearly identified, that the number of cases with not T-staging (white glyphs) is much higher for male patients as for female. There was no hypothesis to explain this difference. Further investigation explained this as wrong classification, as most of thw cases included a secondary finding about a colon tissue, which is done in combination with a prostate biopsy.

**Figure 11:**
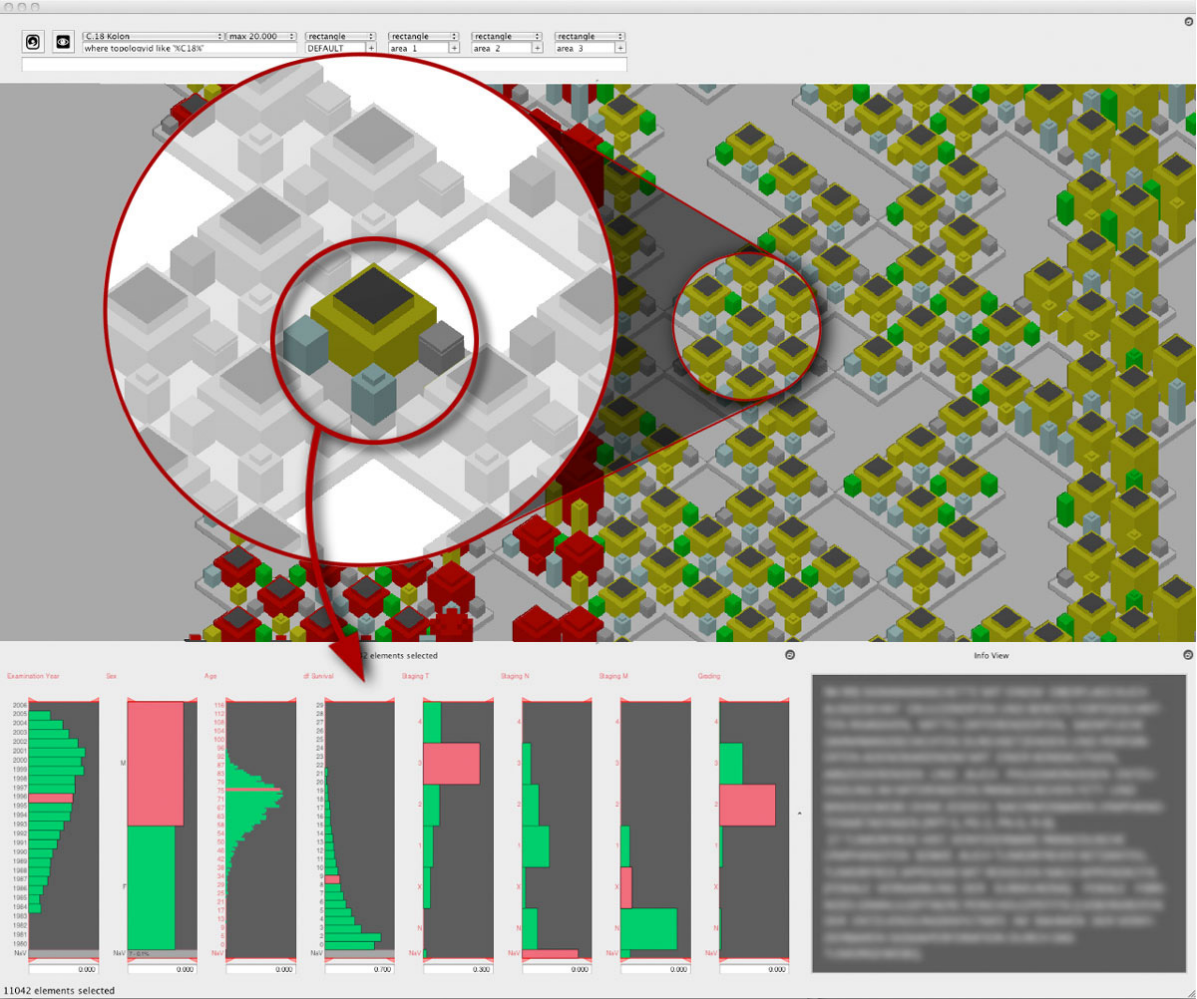
**Colon cancer cases, manually grouped**. The visualization is done with detail glyphs (figure11.png). Further magnification shows the glyphs in the detail view. The user can compare the N-staging, M-staging and the grading for a small number of glyphs. The *disease analyser *depicts the variable values of the current selected element in the histogram view. Additionally the full text diagnosis of the selected element is shown in a text window.

A further zoom-in shows the glyphs in the detail view, see Figure 11. The user can now compare the N-staging, M-staging and the grading for a small number of glyphs. The disease analyser shows the variable values of the current selected element in the histogram view and the full text diagnosis is shown in a text window on the right side (blurred for anonymisation). Here the disease analyser is used to manually select and compose subgroups for clinical studies. In our example two subgroups of colon cancer tissues were selected, by maximum difference in grading and disease free survival together with a preferably complete follow up diagnosis.

## Discussion

The utilization of multilevel data glyphs in the disease analyser was a valuable source for the development of our glyph design criteria. In the design process we faced the following challenges:

• *Occlusion*: 3D glyphs provide on the one hand high data density, but on the other hand face the problem of occlusion. To minimize the occlusion effect we put the main visual variable on top of the geometry (especially in the iconic view) and limit the height of the data glyph. Perspective distortions are avoided by the use a parallel projection (2½D view of an object with forced depth). We use either a dimetric projection or a cavalier or military projection when the glyphs should be seen from a higher point of view.

• *Secondary colors: *Multilevel glyphs consist of complex geometry, where each geometric primitive can be colored independently. This may result in undesirable secondary (mixed) colors. To avoid this effect a good glyph design provides a clear gradation of visual variables, especially for color perception. Such a gradation can be achieved through well defined increments of the graphic primitives size and a restricted color mapping for individual graphical primitives. In some special cases secondary colors could be used intentionally, e.g. to visualize the coincidence of two values in a large data set.

• *Grid patterns*: When data glyphs are arranged in a dense grid unwanted patterns can occur. To avoid this, a good glyph design is based on a symmetrical skeletal structure. Especially in the iconic view it is crucial to model borders of the glyph, in order to provide a good visual differentiation. In the simplest case a border can be realized through a plinth as a neutral base element.

During beta testing the disease analyser was used by 12 experts working in the field of bioinformatics, computational biology and medical research. The first group had a focus on data acquisition, automatic classification of medical records and data quality issues. The focus of the second group was on data analysis, e.g. the development of the health care system, and hypothesis generation. The following observations and statements describe their experience and provide valuable input for further developments:

• The disease analyser is very well suited to find outliers and "white spaces" in the source data.

• Snapshot and bookmarking functionality is missing.

• The selection of subgroups within the histograms and the visual comparison of the value distributions were very much appreciated.

• In research tasks, the disease analyser was used to compare two to four subgroups.

• Manual arrangement and sorting of cases was used often.

• The fast availability of the full diagnosis text for the selected data glyph is an important feature.

• When a hypothesis is generated there should be a report module to (statistically) compare the involved subgroups and to print out a report.

## Conclusions

We developed multilevel data glyphs for the visualization of large medical data sets. The data glyphs provide

• three levels of detail (semantic zoom) suitable for a different screen space, and a

• validation of the data variable mapping.

We used multilevel data glyphs in the *disease analyser*, a visual analytic application for quality control and exploration of a comprehensive collection of cancer disease records. Three concrete glyph designs and design rules resulted out of the hands-on- experience.

We plan to integrate the proposed data glyphs as a visual front end to the biobank of the Medical University Graz and for quality assurance tasks of data record related to cancer samples and to apply the visualization method for strategic planning and trend analysis in the medical domain. In the undertaking we will use a lightweight (webGL) version of data glyphs, which can be used as visualization components in a webpage connected to a local datagrid or through a web service to a central medical database.

There are a lot of studies to compare of 2D versus 3D visualization techniques for the visualizations of spatial related data, e.g. medical renderings or geographic data. However there is now systematic evaluation known to the authors comparing 2D glyphs to 3D and 2½D (isometric) techniques for abstract information. For abstract information no inherent mapping of the data either to the 3D shape of a glyph nor the spatial position is given, which would be a natural mental model for users of the visualization results. Lie et al [38] have discussed design and realization aspects (occlusion, depth perception and visual cluttering) of glyph based 3D-data visualization with a focus on glyph placement. Their work is a good starting point for a systematic evaluation of the shape/placement of 2½D glyphs providing high data density versus 2D shapes, which are less challenging for the user perception.

A second open research question is how to build and evaluate smooth transitions between different levels of glyph abstraction. In the current work the glyph rendering method was changed due to the glyph size in the screen space. The configuration of "switching points" was done with a heuristic approach, and carefully (manual) designed glyph geometry resulted in a smooth visual transition. However a systematic study and description of the methodology of glyph transitions (fusion of semantic and graphical zoom) has still to be done.

## Competing interests

The authors declare that they have no competing interests.

## Authors' contributions

HM and KZ conceived the idea in the analysis of the pathological finding database of the Medical University Graz. HM developed the medical glyph concept and defined together with KZ and AH medical needs and usability criteria. The OpenGL/C++ application was written by HM and RR. The database was administered by RR. All authors read and approved the final manuscript.

## Supplementary Material

Additional File 1 **Video of linked sliders (suppl_linked_sliders**.mov). Linked histogram sliders for the selection of subgroups Click here for file

## Declarations

Publication for this article has been funded by the Christian Doppler laboratory for biospecimen research and biobanking technologies.

This article has been published as part of *BMC Bioinformatics *Volume 15 Supplement 6, 2014: Knowledge Discovery and Interactive Data Mining in Bioinformatics. The full contents of the supplement are available online at http://www.biomedcentral.com/bmcbioinformatics/supplements/15/S6.
